# Oculopharyngeal muscular dystrophy mutations link the RNA‐binding protein HNRNPQ to autophagosome biogenesis

**DOI:** 10.1111/acel.13949

**Published:** 2023-08-09

**Authors:** Hasan Ishtayeh, Margarita Galves, Tania T. Barnatan, Yevgeny Berdichevsky, Fatima Amer‐Sarsour, Metsada Pasmanik‐Chor, Itzhak Braverman, Sergiu C. Blumen, Avraham Ashkenazi

**Affiliations:** ^1^ The Department of Cell and Developmental Biology, Faculty of Medicine Tel Aviv University Tel Aviv Israel; ^2^ Bioinformatics Unit, G.S. Wise Faculty of Life Science Tel Aviv University Tel Aviv Israel; ^3^ Department of Otolaryngology, Head and Neck Surgery Hillel Yaffe Medical Center Hadera Israel; ^4^ Rappaport Faculty of Medicine, Technion Haifa Israel; ^5^ Department of Neurology Hillel Yaffe Medical Center Hadera Israel; ^6^ Sagol School of Neuroscience Tel Aviv University Tel Aviv Israel

**Keywords:** autophagy, autophagy genes, nutrient deprivation, trinucleotide repeat expansion

## Abstract

Autophagy is an intracellular degradative process with an important role in cellular homeostasis. Here, we show that the RNA binding protein (RBP), heterogeneous nuclear ribonucleoprotein Q (HNRNPQ)/SYNCRIP is required to stimulate early events in autophagosome biogenesis, in particular the induction of VPS34 kinase by ULK1‐mediated beclin 1 phosphorylation. The RBPs HNRNPQ and poly(A) binding protein nuclear 1 (PABPN1) form a regulatory network that controls the turnover of distinct autophagy‐related (ATG) proteins. We also show that oculopharyngeal muscular dystrophy (OPMD) mutations engender a switch from autophagosome stimulation to autophagosome inhibition by impairing PABPN1 and HNRNPQ control of the level of ULK1. The overexpression of HNRNPQ in OPMD patient‐derived cells rescues the defective autophagy in these cells. Our data reveal a regulatory mechanism of autophagy induction that is compromised by PABPN1 disease mutations, and may thus further contribute to their deleterious effects.

## Graphical abstract

Autophagy is an important degradation pathway to protect cells from stress conditions in aging and age‐related diseases. Here, we uncover regulatory pathways of starvation‐induced autophagy by the RNA‐binding proteins, heterogeneous nuclear ribonucleoprotein Q (HNRNPQ) and poly(A) binding protein nuclear 1 (PABPN1), and showed that these pathways are perturbed by oculopharyngeal muscular dystrophy (OPMD) mutations.

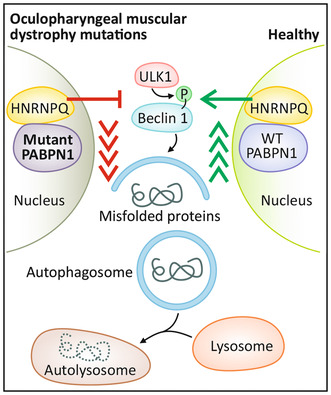

**Abbreviations**
ATG: autophagy‐related
BafA1: bafilomycin A1
HNRNPQ: heterogeneous nuclear ribonucleoprotein Q
LC3: microtubule‐associated protein 1A/1B‐light chain 3
OPMD: oculopharyngeal muscular dystrophy
PABPN1: poly(A) binding protein nuclear 1
RBP: RNA‐binding protein
ULK1: Unc‐51 like autophagy activating kinase 1

## 1 INTRODUCTION

Macroautophagy (henceforth autophagy) is responsible for transferring cytoplasmic materials to the lysosomes for degradation. As a result, autophagy sustains cellular renovation and homeostasis by recycling molecular building blocks (such as amino acids or fatty acids) for anabolic processing (Bento et al., 2016; Boya et al., 2013; Dikic & Elazar, 2018; Levine & Kroemer, 2019). The first morphologically recognizable autophagic precursor is a double‐membraned structure called a phagophore, whose edges elongate and fuse while engulfing a portion of the cytoplasm. This produces a spherical double‐membrane organelle, called the autophagosome (Bento et al., 2016; Dooley et al., 2014; Mizushima et al., 2011). The formation of autophagosomes requires several steps (nucleation, elongation, and closure), which are regulated by conserved proteins termed autophagy‐related (ATG) proteins (Mizushima et al., 2011). During autophagosome formation, members of the ATG8 ubiquitin‐like family of proteins are conjugated to the lipid phosphatidylethanolamine (PE) in autophagosomal membranes (Kabeya et al., 2000; Nguyen & Lazarou, 2022). As a result, measuring the LC3 lipidation relatively to a loading control, scoring the number of LC3 vesicles, and detecting the degradation of autophagy substrates are the mainstay methods used to monitor autophagy (Klionsky, 2016).

The basic function of eukaryote‐conserved process of autophagy is to protect cells against nutrient deprivation (starvation) and related stresses (Alirezaei et al., 2010; Boya et al., 2013; Das et al., 2012; Hewitt & Korolchuk, 2017; Kuma et al., 2004). Upregulation of autophagy in response to these conditions allows cells to degrade macromolecules to their building blocks, which can then be re‐used to provide energy (Kuma et al., 2004). In addition, autophagy is also part of the protein quality control mechanisms that ensure the degradation of toxic aggregate‐prone proteins, such those with polyglutamine and polyalanine‐expansion mutations (Ashkenazi et al., 2017; Ravikumar et al., 2002; Scrivo et al., 2018; Valionyte et al., 2020).

The RNA‐binding protein (RBP), poly(A) binding protein nuclear 1 (PABPN1) contains an N‐terminal polyalanine tract with up to 10 alanine residues, but an expansion mutation of the polyalanine tract in PABPN1 (leading to a stretch of 11–18 alanines), causes a specific pathology, termed oculopharyngeal muscular dystrophy (OPMD; Brais et al., 1998; Robinson et al., 2005). OPMD is a late‐onset progressive autosomal dominant disease that generally presents in the fifth or sixth decade of life with dysphagia, ptosis, and proximal limb weakness (Blumen et al., 1993; 2009; Richard et al., 2017). While OPMD has a world‐wide distribution, it is more prevalent among French‐Canadians and the Jews from Uzbekistan (Blumen et al., 1993; Hill et al., 2001; Richard et al., 2017). A pathological hallmark of mutant PABPN1 with an expanded polyalanine tract is the formation of nuclear aggregates (Brais et al., 1998; Davies et al., 2006). Studies of OPMD mouse models suggest that the loss of PABPN1 is not sufficient to mimic the entire disease expansion pathology (Vest et al., 2017). One protein that interacts with PABPN1 is the RBP, heterogeneous nuclear ribonucleoprotein Q (HNRNPQ), which is also known as SYNCRIP (Banerjee et al., 2019). HNRNPQ is a member of the HNRNP RNA‐binding proteins in mammalian cells, which are involved in stabilization of mRNA, and transcriptional, and translational regulation (Geuens et al., 2016). HNRNPQ has been shown to suppress polyglutamine expanded huntingtin aggregation (Ryu et al., 2019), suggesting it may have a role in protecting cells from stress, but this remains unknown.

Here, we elucidate the functions of HNRNPQ in autophagy and use the autophagy induction response in OPMD cell models and patient‐derived cells to demonstrate that HNRNPQ cooperates with PABPN1 to regulate early events in autophagosome biogenesis. In addition, the results reveal previously undescribed regulators of Unc‐51 like autophagy activating kinase 1 (ULK1), which is a critical factor in autophagy induction and termination (Allavena et al., 2016; Liu et al., 2016; Nazio et al., 2016; Russell et al., 2013). Importantly, these regulators are perturbed by disease‐causing mutations.

## 2 RESULTS

### 2.1 HNRNPQ contributes to autophagosome biogenesis

**FIGURE 1:**
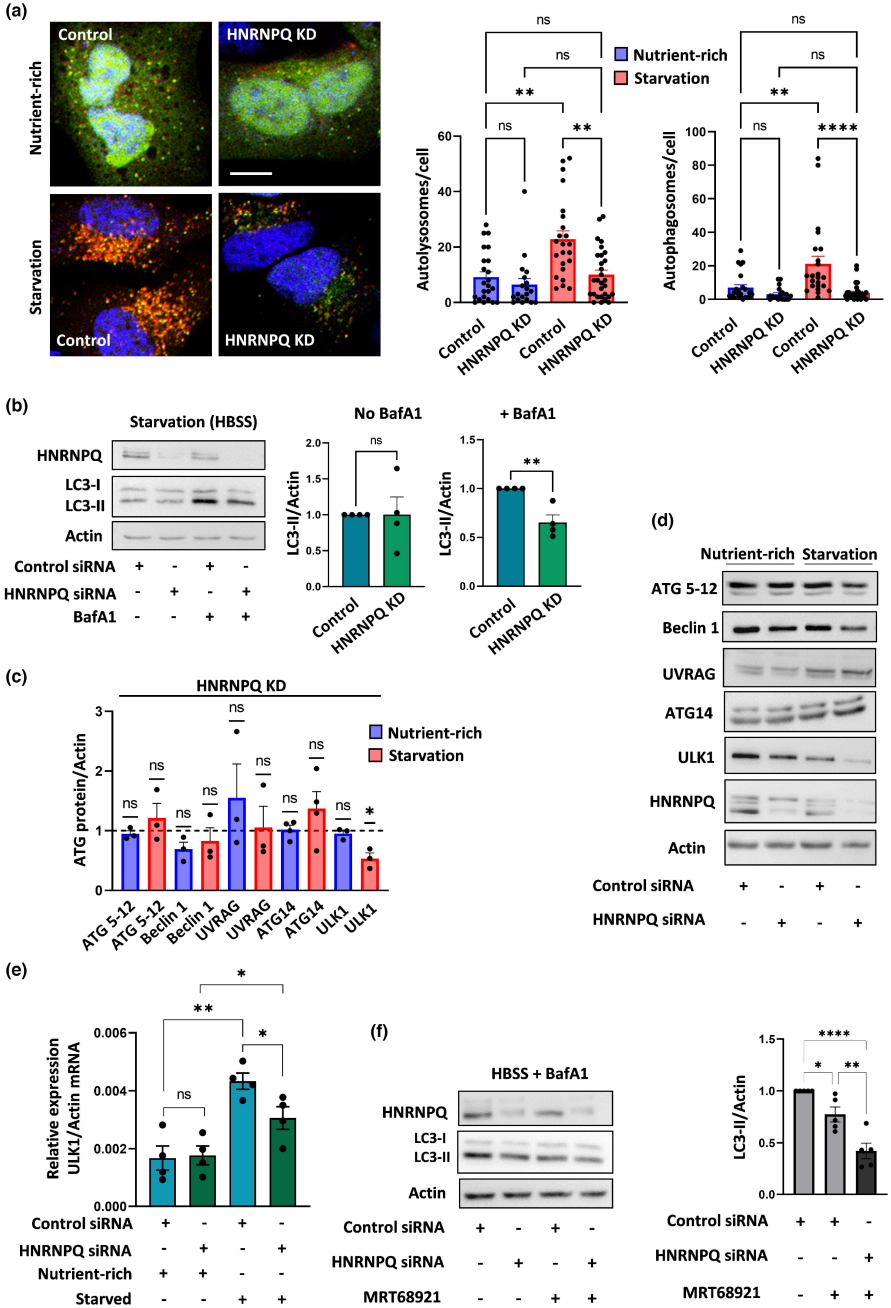
Heterogeneous nuclear ribonucleoprotein Q (HNRNPQ) contributes to autophagosome formation and stabilizes ULK1 levels. (a) HeLa cells were treated with siRNA targeting HNRNPQ or control siRNA and transfected with mRFP‐GFP‐LC3 plasmid. The cells were kept in nutrient‐rich media or starved (HBSS, 4 h) before image analysis. Representative images of mRFP‐GFP‐LC3 puncta in cells used to score the number of autolysosomes (mRFP only puncta) and autophagosomes (RFP + GFP puncta). Scale bar 10 μm. Results were quantified as the difference in the number of autolysosomes and autophagosomes in control and HNRNPQ knockdown (KD) cells. Each dot on the bar chart represents the total number of vesicles per cell. Data are representative from one out of three independent experiments. One‐way ANOVA Kruskal–Wallis test. (b) Control and HNRNPQ KD cells were starved with or without Bafilomycin A1 (BafA1) (250 nM, 4 h), and cell lysates were analyzed for LC3‐II levels. Results (n = 4 experiments) are normalized to control cells. Paired two‐tailed t test. (c) HNRNPQ KD and control cells were kept in nutrient‐rich media or starved, and cell lysates were screened for various autophagy‐related (ATG) proteins involved in autophagy induction. ATG protein levels are normalized to actin. The dotted line at y = 1 represents the control value to which each protein levels is compared in the HNRNPQ KD cells. At least three independent experiments were used for analysis. Paired two‐tailed t test. (d) Representative western blots showing the levels of the ATG proteins in the control and KD cells. (e) Analysis of ULK1 mRNA levels by qRT‐PCR in control and HNRNPQ KD cells under nutrient‐rich and starvation conditions: N = 4 experiments. Unpaired two‐tailed t test. (f) Control and HNRNPQ KD HeLa cells were treated with BafA1 in starvation media with or without MRT68921 (10 μM in HBSS, 4 h), and lysates were analyzed for LC3‐II levels: N = 5 experiments, one‐way ANOVA Holm–Sidak test. * *p* < 0.05, ** *p* < 0.01, *** *p* < 0.001, **** *p* < 0.0001, ns is non‐significant.

In order to investigate the function of HNRNPQ in autophagy, we utilized the dual fluorescent tag, GFP‐mRFP conjugated to the autophagy‐related microtubule‐associated proteins 1A/1B light chain 3B (termed LC3; Ashkenazi et al., 2017; Kimura et al., 2007; Klionsky, 2016). Autophagy induction by nutrient deprivation (4 h starvation in HBSS) in control siRNA‐treated HeLa cells expressing the GFP‐mRFP LC3 vector, increased the number of yellow puncta (GFF positive/mRFP positive; Figure 1a). These puncta turn red (GFP negative/mRFP positive) upon fusion of an autophagosome with a lysosome. In contrast, starved HNRNPQ‐depleted cells (using siRNA targeting HNRNPQ) did not show a significant increase in the number of autophagosomes (yellow puncta). This was accompanied by a lower number of autolysosomes (red puncta) in the starved HNRNPQ‐depleted cells as compared to starved control cells (Figure 1a). The results suggest that autophagy flux is decreased in HNRNPQ‐depleted cells, in particular when induced by starvation. Next, we treated the HNRNPQ‐depleted and control HeLa cells with Bafilomycin A1 (BafA1) and analyzed the LC3‐II levels relative to a loading control. Since BafA1 poisons the lysosomes, LC3‐II levels in the BafA1‐treated cells reflect the production rate of autophagosomes (Klionsky, 2016). Under BafA1 treatment, the levels of LC3‐II in HNRNPQ‐depleted cells were decreased compared to control cells under nutrient‐rich condition and were further decreased by starvation (Figure 1b, Figure S1a). Moreover, this decrease in LC3‐II levels in the BafA1 treated cells was validated by three independent HNRNPQ targeting siRNAs (Figure S1b). These results suggest that HNRNPQ contributes to autophagosome synthesis.

### 2.2 HNRNPQ regulates starvation‐dependent ULK1 turnover

The mechanisms by which HNRNPQ affects autophagosome formation were investigated by screening HNRNPQ‐depleted cells under nutrient‐rich and deprived conditions for various ATG proteins, and comparing the levels to those in control cells (Figure 1c). These included key ATG proteins involved in the initiation of autophagosome biogenesis in response to starvation. The results indicated that the levels of ULK1 protein are significantly reduced in starved HNRNPQ‐depleted cells compared to controls, although no differences were detected in a nutrient‐rich environment (Figure 1c,d). In contrast, starved cells overexpressing HNRNPQ showed increased ULK1 levels (Figure S1c). This may be related to the observation that ULK1 is a key inducer of autophagy initiation and autophagosome biogenesis whose activity is stimulated in nutrient‐deprived cells (Allavena et al., 2016; Bento et al., 2016; Dikic & Elazar, 2018; Ktistakis & Tooze, 2016; Liu et al., 2016; Nazio et al., 2016; Russell et al., 2013). Since the rate of ULK1 turnover is tightly regulated in order to control the induction and termination of autophagy in response to stress (Allavena et al., 2016; Liu et al., 2016; Nazio et al., 2016; Russell et al., 2013), we analyzed ULK1 mRNA levels in the siRNA‐control and HNRNPQ‐depleted cells (Figure 1e). The results revealed that ULK1 mRNA levels were induced by starvation but to a lesser extent in HNRNPQ KD cells than in the controls, suggesting that HNRNPQ is a positive regulator of ULK1 mRNA expression (Figure 1e). Moreover, there was no change in the cellular location of the cytoplasmic and nuclear isoforms of HNRNPQ when autophagy was induced by starvation (Figure S1d).

The ULK1 inhibitor, MRT68921, was used to examine whether the effects of HNRNPQ on autophagy are entirely dependent on ULK1. This inhibitor specifically blocks the autophagic flux, as indicated by a reduction in LC3‐II levels in HeLa cells treated with the inhibitor together with HBSS and BafA1 (Petherick et al., 2015; Figure 1f). Interestingly, we observed a greater reduction in LC3‐II levels after MRT68921‐treatment in the HNRNPQ‐depleted cells than in control cells (Figure 1f). This suggests the possibility that autophagy signaling is affected by HNRNPQ by other mechanisms in addition to regulating ULK1 levels.

### 2.3 PABPN1 is a positive regulator of autophagy induction and ULK1 mRNA levels

**FIGURE 2:**
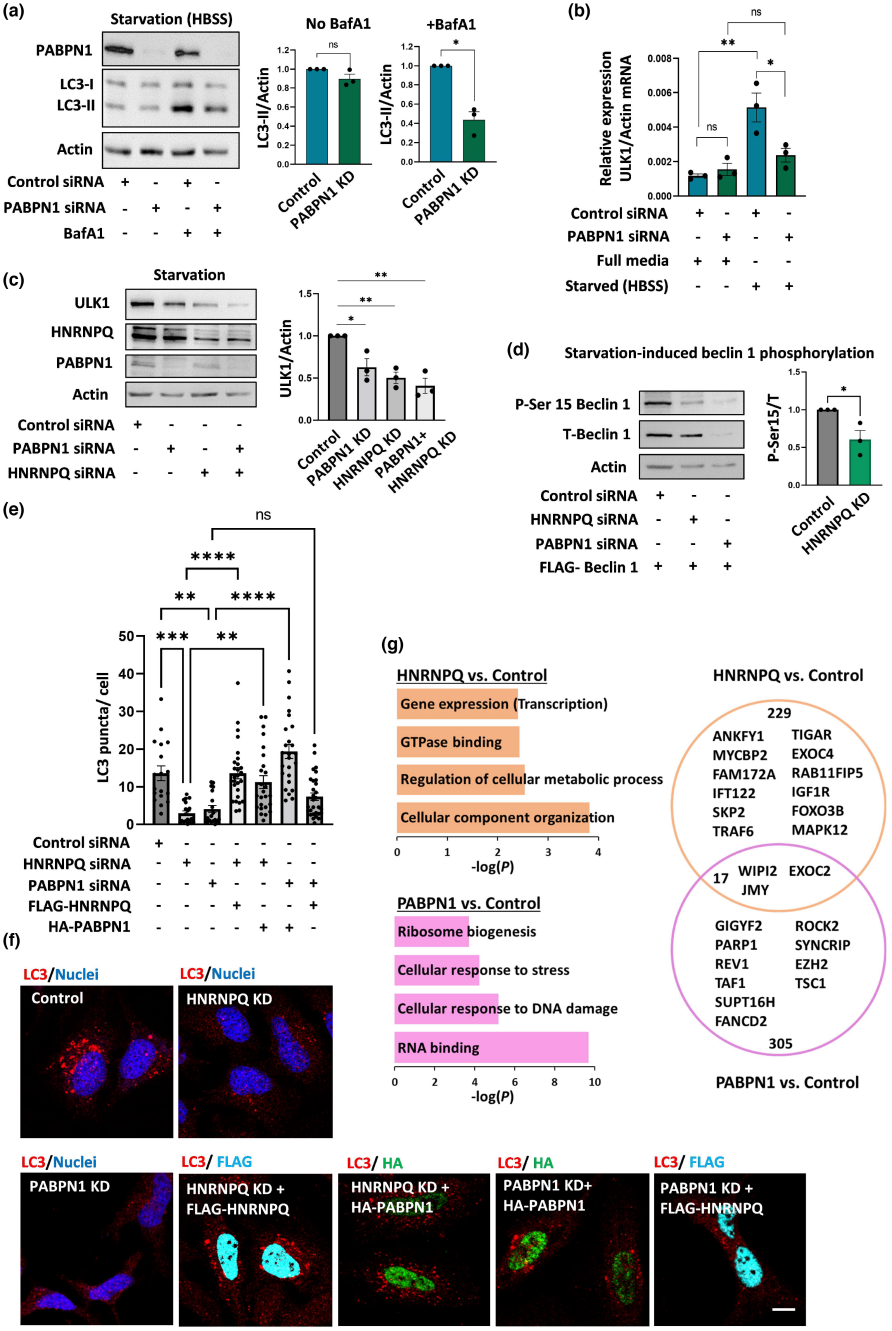
Poly(A) binding protein nuclear 1 (PABPN1) regulates the effects of heterogeneous nuclear ribonucleoprotein Q (HNRNPQ) on autophagy involving ULK1 and beclin 1. (a) HeLa cells were treated with siRNA targeting PABPN1 or control siRNA. The cells were starved with or without exposure to BafA1 (250 nM in HBSS for 4 h). Cell lysates were analyzed for LC3‐II levels. Results are normalized to control cells: n = 3 experiments, paired two‐tailed t test. (b) Analysis of ULK1 mRNA levels by qRT‐PCR in control and PABPN1 KD cells under nutrient‐rich and starvation conditions: n = 3 experiments. Unpaired two‐tailed t test. (c) Detection of ULK1 protein levels in starved control cells, HNRNPQ KD cells, PABPN1 KD cells, and in double HNRNPQ and PABPN1 KD cells: n = 3 experiments, paired two‐tailed t test. (d) Control, HNRNPQ KD, and PABPN1 KD cells were transfected with FLAG‐Beclin 1 plasmid and starved (HBSS, 4 h). Cell lysates were analyzed for beclin 1 phosphorylation on Ser 15. The ratio of phosphorylated to total beclin 1 is presented. Results were normalized to control cells: n = 3 experiments, paired two‐tailed t test. (e) Control, HNRNPQ KD, and PABPN1 KD cells were transfected with empty vector or FLAG‐HNRNPQ (colored magenta) or HA‐PABPN1 (colored green). The cells were starved and imaged for endogenous LC3 puncta (colored red). The numbers of LC3 puncta per cell in different image fields from three independent experiments are presented. One‐way ANOVA Kruskal–Wallis test. (f) Representative images of endogenous LC3 puncta under the different conditions. Scale bar 10 μm. (g) Starved HeLa cells were lysed and subjected to RNA immunoprecipitation with HNRNPQ or PABPN1 or control unrelated antibodies. RNA was isolated from the immunocomplexes and analyzed by RNA‐seq. Pathways enriched (−log*P* >2) in the brown module (HNRNPQ vs. control) and in the pink module (PABPN1 vs. control) are presented as well as a Venn diagram of the number of genes from the brown module that either overlap or are distinct from gene members in the pink module. The diagram also contains a number of representative autophagy‐regulating genes. *p < 0.05, **p < 0.01, ***p < 0.001, ****p < 0.0001, ns is non‐significant.

As one such possible HNRNPQ‐related autophagy signaling pathway, we focused on the RBP, PABPN1 which is an interactor of HNRNPQ (Banerjee et al., 2019). Treating PABPN1 depleted cells with BafA1 decreased the levels of LC3‐II under both nutrient‐rich and starvation conditions (Figure 2a, Figure S2a,b) compared to control HeLa cells. This was accompanied by an impaired elevation of ULK1 mRNA levels and a decrease in ULK1 protein levels after starvation (Figure 2b, Figure S2c). In order to examine the relationship between the two RBPs, PABPN1 and HNRNPQ on regulating ULK1 levels, we conducted single and double knockdown (KD) experiments (Figure 2c). We observed that the depletion of one RBP did not decrease the protein levels of the other (Figure S2d), nor did it cause a significant change in total protein levels (Figure S2e). Interestingly, cells with HNRNPQ KD or PABPN1 KD showed reduced ULK1 protein levels, and the double KD did not reduce the levels of ULK1 further (Figure 2c).

### 2.4 Effects of PABPN1 and HNRNPQ on the phosphorylation and levels of beclin 1 complexes

ULK1‐mediated phosphorylation of serine 15 (Ser 15) in beclin 1 is required for stimulating VPS34 kinase activity and for full autophagy induction by starvation (Russell et al., 2013). We used a specific antibody to detect Ser 15 phosphorylation when FLAG‐tagged beclin 1 is overexpressed in the cells (Figure 2d). HNRNPQ‐depleted cells exhibited a decrease in beclin 1 Ser 15 phosphorylation, which is consistent with the lower levels of ULK1. Notably, beclin 1 phosphorylation was almost undetectable in the PABPN1‐depleted cells where the total levels of the FLAG‐beclin 1 were markedly reduced (Figure 2d). Since beclin 1 is generally found in complexes with other ATG proteins, such as ATG14 and UVRAG (Itakura et al., 2008), we assessed whether depletion of HNRNPQ or PABPN1 affects the composition of the beclin 1 complexes, thereby further contributing to the impaired autophagy initiation phenotype. The results presented in Figure 1c,d and Figure S3a‐d indicate that HNRNPQ depletion does not reduce the protein or mRNA levels of ATG14 or UVRAG under nutrient‐rich or starvation conditions. In contrast, while PABPN1 depletion did not affect ATG14 or UVRAG at the mRNA level, there was a reduction in the protein levels (Figure S3e). This suggests that PABPN1 may regulate the turnover of beclin 1 complexes to control autophagosome biogenesis. Since the members of the beclin 1 complex are mutually dependent for stability (Liu et al., 2011), the decrease in beclin 1 levels detected after PABPN1 depletion may destabilize the levels of the ATG14 and UVRAG proteins. PABPN1 regulates alternative polyadenylation (APA) in the 3′ untranslated region (UTR) of targeted RNA (Jenal et al., 2012), which has been suggested to affect the translation of several ATG mRNA into proteins (Raz et al., 2017). Although APA regulation requires two polyadenylation signals at the 3’ UTR, our analysis of the ULK1 RNA sequence detected only one polyadenylation signal (“aataaa”) at the ULK1 3’ UTR. This implies that the PABPN1 influence on ULK1 mRNA levels may be distinct from APA activity and may also involve the regulation of other RBPs that are associated with PABPN1.

### 2.5 Networks of autophagy‐regulating genes related to HNRNPQ and PABPN1

As the next stage, we investigated the relationships between PABPN1 and HNRNPQ with regard to the autophagy phenotypes. For this purpose, we utilized vectors expressing the HNRNPQ nuclear isoform, or expressing PABPN1, to conduct autophagy rescue experiments in HNRNPQ KD and PABPN1 KD cells (Figure 2e,f). We did not use RNAi resistant vectors since there was sufficient level of expression. Immunofluorescence analysis of the positive cells showed that the expression rates of FLAG‐HNRNPQ in HNRNPQ KD cells and HA‐PABPN1 in PABPN1 KD cells were 46% and 73%, respectively. In accordance with the reduction seen in LC3‐II levels, starved cells depleted for HNRNPQ or PABPN1 also exhibited a lower number of LC3 puncta than control cells, which correlates with the reduced autophagosome load in the KD cells (Figure 2e,f). Transfection with nuclear HNRNPQ fully restored the number of LC3 puncta in the HNRNPQ‐depleted cells but not in PABPN1 KD cells (Figure 2e,f). In contrast, the number of LC3 puncta were fully restored by the transfection of PABPN1 to cells depleted of either HNRNPQ or PABPN1 (Figure 2e,f). These results suggest that PABPN1 and HNRNPQ affect common autophagy‐regulating pathways (as described above for the ULK1‐beclin 1 axis) but may also regulate other pathways independently.

In order to address this hypothesis, we used RNA immunoprecipitation (RIP) to map the global mRNA species that interact with endogenous HNRNPQ and PABPN1 in the starved HeLa cells (Figure 2g, Table S1). Pathway enrichment analyses of the brown module (246 genes enriched in the HNRNPQ pulldown compared to control) revealed categories enriched for cellular component organization and metabolic processes. Other enriched pathways detected are related to GTPase binding and regulation of transcription (Figure 2g). The pathway enrichment analyses of the pink module (322 genes enriched in PABPN1 pulldown compared to control) revealed distinct pathways from those in the brown module. The observation that these involve the cellular response to DNA damage supports the validity of our analysis, since PABPN1 has previously been reported to be required for the DNA damage response (Gavish‐Izakson et al., 2018). Interestingly, we identified both shared and distinct genes that were enriched in the brown and pink modules, further supporting our initial hypothesis of the regulation of both common and discrete pathways by HNRNPQ and PABPN1, some of which could be related to autophagy. This can be visualized by the Venn diagram in Figure 2g, which presents selected genes known to affect autophagy. Since we could not detect ULK1 in our RIP setting, our current model supports the indirect regulation of ULK1 mRNA levels by HNRNPQ and PABPN1, possibly by an effect on ULK1 transcriptional programs. Moreover, we cannot exclude the possibility that HNRNPQ regulates other RBPs that are important to stabilize ULK1 mRNA levels.

### 2.6 Mutant PABPN1 associates with HNRNPQ and impairs ULK1

**FIGURE 3:**
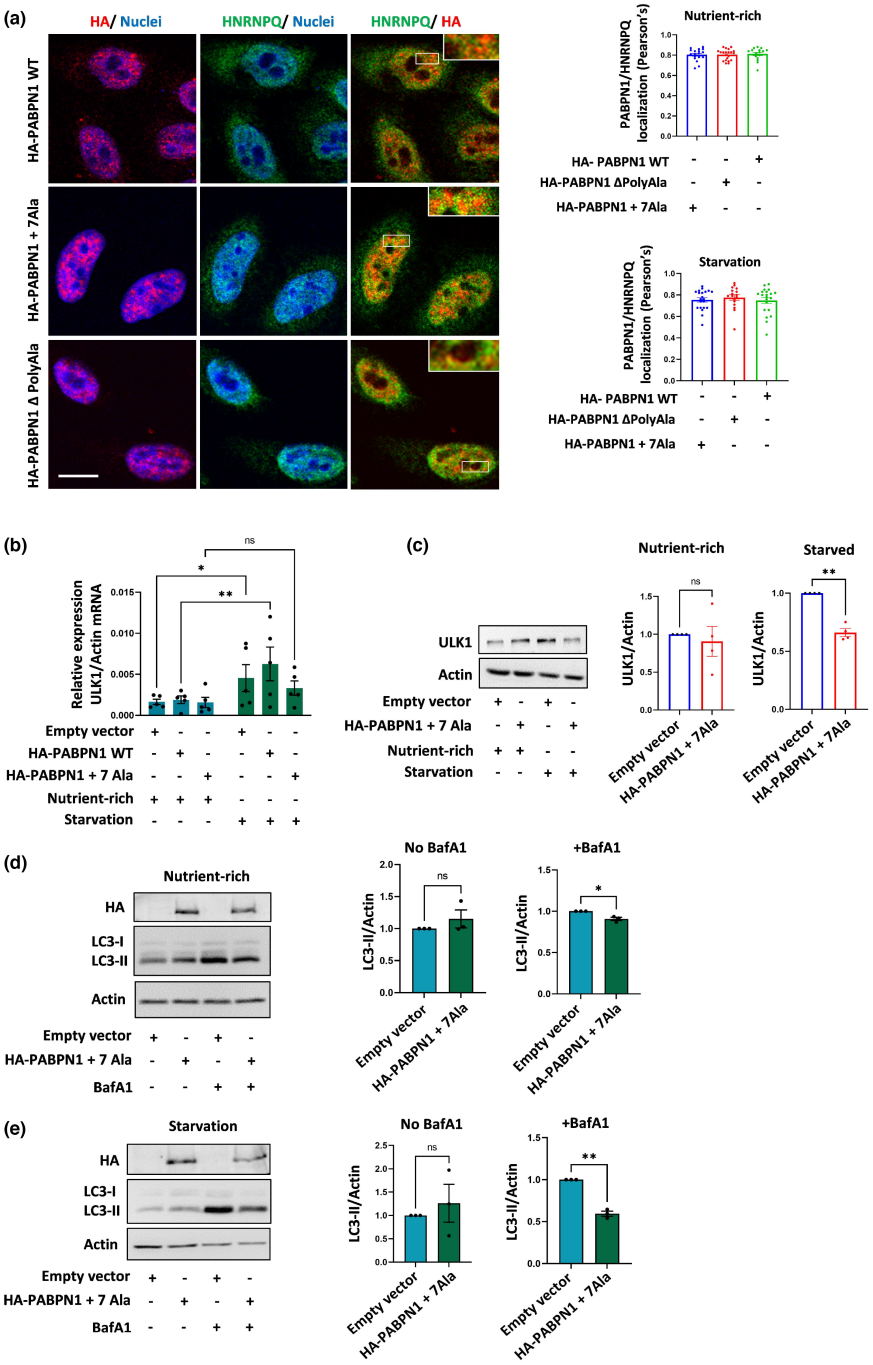
Mutant poly(A) binding protein nuclear 1 (PABPN1) associates with heterogeneous nuclear ribonucleoprotein Q (HNRNPQ), impairs starvation‐induced autophagy, and reduces ULK1 levels. (a) The following vectors were expressed in HeLa cells: HA‐PABPN1 wild‐type (WT), HA‐PABPN1 mutant (+7Ala), and HA‐PABPN1 with a deletion of the polyalanine domain (PABPN1 ΔPolyAla). The cells were maintained in nutrient‐rich media or starved (HBSS, 4 h), and imaged for PABPN1 (HA staining colored red) and endogenous HNRNPQ (colored green). Quantification of the association of HA‐PABPN1 mutants with endogenous HNRNPQ (Pearson's coefficient). Results are the average values from cells in different imaged fields (approximately 50 cells for each condition). Images of the different conditions are shown in the cells maintained in nutrient‐rich condition. Scale bar 10 μm. (b) Analysis of ULK1 mRNA levels by qRT‐PCR in cells transfected with empty vector, HA‐PABPN1 WT and HA‐PABPN1 mutant (+7Ala) under nutrient‐rich and starvation conditions: n = 5 experiments. Unpaired two‐tailed t test. (c) HeLa cells were transfected with empty vector and HA‐PABPN1 mut (+7Ala) and were maintained in nutrient‐rich or starvation conditions before being analyzed for ULK1 protein levels. Results are normalized to control cells: n = 4 experiments, paired two‐tailed t test. (d, e) Empty vector and HA‐PABPN1 mutant (+7Ala) transfected cells were maintained in nutrient‐rich condition or starved with or without BafA1 (250 nM, 4 h), and cell lysates were analyzed for LC3‐II levels. Results (n = 3 experiments) are normalized to control cells. Paired two‐tailed t test. * *p* < 0.05, ** *p* < 0.01, ns is non‐significant.

Next, we examined the effect of the disease‐causing polyalanine expansion mutation in PABPN1 on the association with HNRNPQ and autophagy phenotypes. To this end, we expressed the wild‐type and disease‐causing PABPN1 with an expansion mutation of +7 alanine residues (mut PABPN1 + 7 Ala) in HeLa cells. Imaging the cells at low laser intensity to assess differences between wild‐type and mutant PABPN1 nuclear staining revealed wild‐type PABPN1 as both small puncta and diffused patterns. In contrast, the mutant PABPN1 formed larger nuclear puncta (Figure 3a). Interestingly, a fraction of the wild‐type and mutant PABPN1 was also present in the cytoplasm under normal conditions but the proteins concentrated primarily in the nucleus after starvation (Figure S4a). Under both nutrient‐rich and starvation conditions, endogenous HNRNPQ colocalized with nuclear wild‐type PABPN1 and also with the disease‐causing mutant protein (Figure 3a). Our results are consistent with previous reports that mutant PABPN1 intranuclear aggregates can cause sequestration of RNA, PABPN1 and other nuclear proteins (Calado et al., 2000; Fan et al., 2003; Jenal et al., 2012). Notably, the association between PABPN1 and HNRNPQ was not decreased by deletion of the polyalanine domain of PABPN1 (Figure 3a). The polyalanine deletion could also form aggregate‐like assemblies in PABPN1 (Klein et al., 2008). This suggests that while the polyalanine expansion mutation can promote the association of HNRNPQ with the nuclear aggregates, it is not critical for the binding to PABPN1.

Because the loss of function of PABPN1 and HNRNPQ reduced ULK1 mRNA levels under starvation conditions, we monitored ULK1 mRNA levels in cells expressing the wild‐type and mutant PABPN1 as a reliable readout for autophagy perturbations in these cells. The results indicated an impaired ULK1 mRNA induction on starvation in the mutant PABPN1 expressing cells that could be correlated with the decreased levels of ULK1 protein (Figure 3b,c). Cells expressing the mutant PABPN1 displayed subtle changes in basal autophagy when compared to empty vector‐expressing control cells with endogenous wild‐type PABPN1 (Figure 3d). However, the mutant PABPN1 expressing cells exhibited marked inhibition of starvation‐induced autophagosome synthesis, as indicated by a reduction in LC3‐II levels in the BafA1 treated cells (Figure 3e). Similar effects were observed between cells overexpressing wild‐type PABPN1 and those expressing the mutant form of the protein (Figure S4b), which is consistent with the autosomal dominant nature of the OPMD mutation.

### 2.7 Restoration of autophagy in OPMD patient cells by expression of HNRNPQ

Primary fibroblasts from an OPMD‐derived patient biopsy and controls that were derived from sex and aged‐matched healthy individuals were cultured to investigate whether the deleterious mechanisms described here could be recapitulated in the patient cells. Since we could not detect intranuclear inclusions visually by staining for PABPN1, we employed biochemical methods of analysis. Patient‐derived fibroblasts exhibited comparable levels of oligomeric forms of PABPN1 compared to control cells (Figure S4c).

**FIGURE 4:**
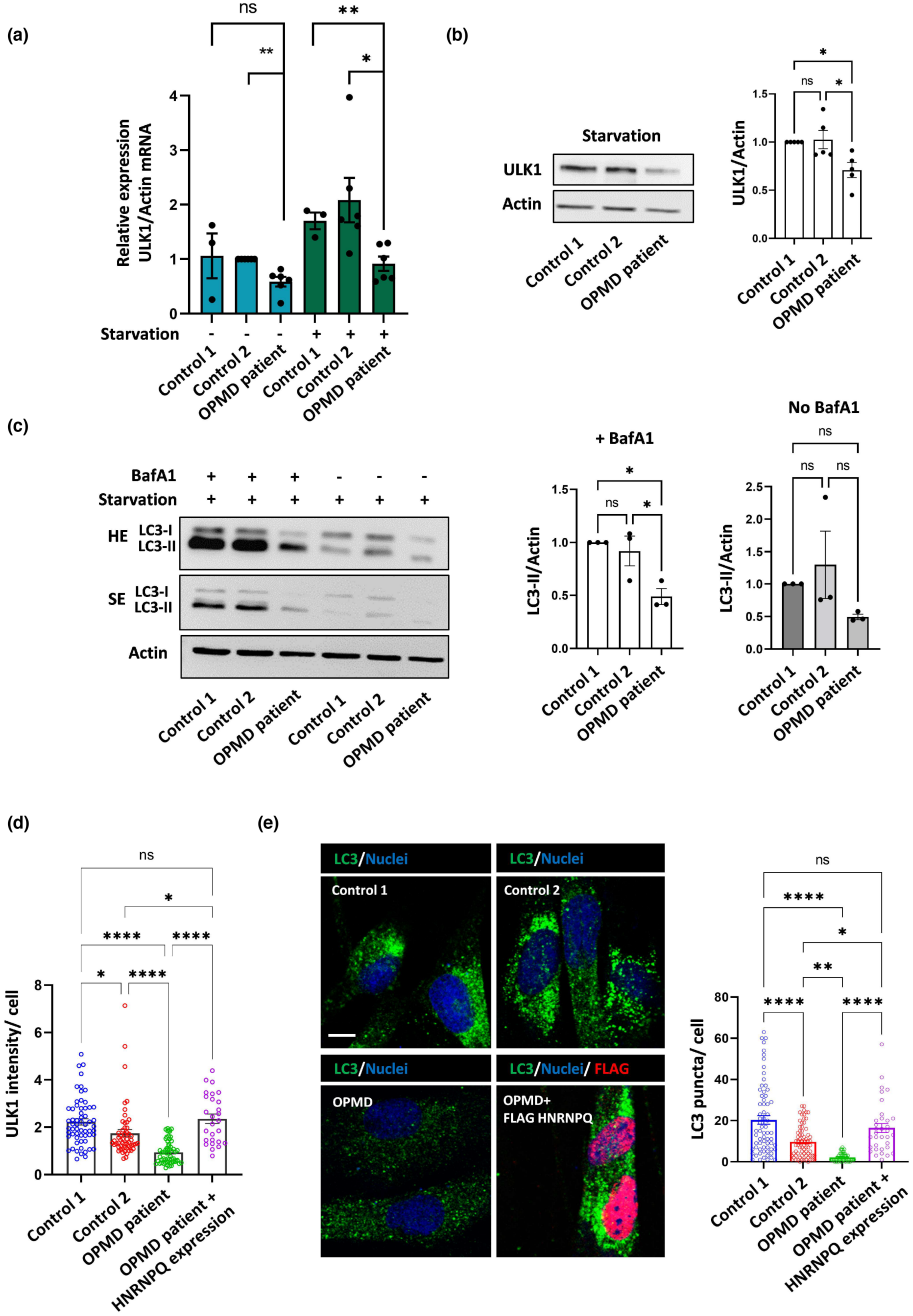
Heterogeneous nuclear ribonucleoprotein Q (HNRNPQ) rescues compromised autophagy in oculopharyngeal muscular dystrophy (OPMD) patient‐derived cells. (a) Primary fibroblasts derived from unaffected individuals (control 1 and control 2) and from an OPMD patient were maintained in nutrient‐rich media or starved (HBSS, 4 h), and the relative expression level of ULK1 mRNA was quantified. Data from at least three independent starvation experiments are presented (in each experiment, the data were normalized to control 2 fed condition). (b) Starved control and OPMD patient fibroblasts were analyzed for ULK1 protein levels. Results are normalized to control fibroblasts. n = 5 experiments. One‐way ANOVA Tukey's test. (c) Control and OPMD patient fibroblasts were starved with or without BafA1 treatment (250 nM, 4 h), and cell lysates were analyzed for LC3‐II levels. SE and HE: short and high exposures. Results (n = 3 experiments) are normalized to control cells. Paired two‐tailed t test. (d, e) OPMD patient fibroblasts were transfected with an empty vector or FLAG‐HNRNPQ. The OPMD patient cells were starved together with control fibroblasts and imaged for endogenous ULK1 intensity (d) and for endogenous LC3 puncta (e). Images of ULK1 analysis are presented in Figure S4g. The different cells and treatments are shown (scale bar 10 μm), as well as the quantification of the numbers of LC3 puncta per from n = 3 experiments. One‐way ANOVA Tukey's test. * *p* < 0.05, ** *p* < 0.01, **** *p* < 0.0001, ns is non‐significant.

Next, we assessed protein aggregation in the control and OPMD patient fibroblasts by using the Proteostat dye, which recognizes aggregates derived from a wide spectrum of protein substrates. The results from normal cells indicated that aggregation increases when the proteasome is inhibited (Figure S4d). Notably, the OPMD patient‐derived cells exhibited more aggregate staining than control cells even with no proteasome inhibition, implying that autophagy is perturbed in the OPMD‐derived fibroblasts. The ULK1 mRNA and protein levels of the patient cells were reduced compared to control cells under nutrient‐rich and starvation conditions (Figure 4a,b, Figure S4e). This was not related to differences in total protein levels between donor‐derived fibroblasts, since there was no significant protein loss in the patient fibroblasts compared to control cells (Figure S4f). Moreover, the decrease in ULK1 levels was accompanied by a decrease in LC3‐II levels in the patient cells treated with BafA1 under starvation condition (Figure 4c). Finally, exogenous expression of HNRNPQ in the patient cells could significantly increase ULK1 protein and LC3 puncta to rescue the defective autophagy in these cells (Figure 4d,e).

## 3 DISCUSSION

Our results reveal the roles of HNRNPQ (SYNCRIP gene) in autophagy and suggest that HNRNPQ is a positive regulator of ULK1 mRNA levels under nutrient deprivation. This then stabilizes ULK1 protein levels and promotes the phosphorylation of beclin 1 to initiate autophagy. Several ATG proteins, including ULK1, are transcriptionally upregulated under starvation conditions (Galves et al., 2023; Nazio et al., 2016). The turnover of ULK1 under nutrient deprivation is not only regulated at the mRNA level, since our results as well as others showed starvation‐dependent decrease in ULK1 protein levels (Allavena et al., 2016; Nazio et al., 2016). Indeed, suppressed translation and increased ULK1 degradation have been proposed as mechanisms to control autophagy termination (Allavena et al., 2016; Liu et al., 2016; Nazio et al., 2016).

Further studies are needed to elucidate how specific transcriptional and mRNA regulation programs, which are mediated by HNRNPQ, impact ULK1 stability. Our RIP analysis has already identified an HNRNPQ‐related module of transcriptional regulation, which may be of interest for future investigation. However, we cannot exclude the possibility of an indirect regulation of ULK1 by HNRNPQ given that both ULK1 and ULK2 are involved in the induction of autophagy (Hara et al., 2008). Moreover, although the focus of this study was on the regulation of the ULK1‐beclin 1 axis, it is clear from our experiments that HNRNPQ has broader regulatory effects on autophagy stimulation (Figure 2c, Figure 2e–g). This is achieved, in part, by the association with other autophagy‐regulating RBPs, exemplified here by PABPN1. Our work, as well as that of others (Banerjee et al., 2019) suggest that PABPN1 can bind HNRNPQ at the protein level. The evidence for an additional association between these two RBPs at the RNA level was provided by our RIP assay where SYNCRIP was identified as a potential target for PABPN1. Such an autophagy‐related cooperation between RBPs is also presented by the finding that unlike wild‐type PABPN1, the disease‐causing protein impairs the HNRNPQ regulation of ULK1.

The autosomal dominant form of OPMD exhibits nuclear aggregates of PABPN1 in muscle tissue (Brais et al., 1998). We found that HNRNPQ localizes with the PABPN1 aggregates (Figure 3a), which can sequester HNRNPQ and thus contributes to compromised autophagy. However, it is likely that nuclear PABPN1 aggregation is not the only mechanism involved in autophagy perturbations. Indeed, we could demonstrate that patient‐derived fibroblasts with mild PABPN1 aggregation capacity, present with autophagy impairment and accumulation of misfolded proteins (Figure 4c–e, Figure S4d). This suggests that perturbations of autophagy may occur in different cell types in OPMD patients.

Our work provides insights into regulation of mammalian autophagy by RBPs, which is an emerging and still understudied field of research. In the context of disease, our results suggest that impaired autophagy induction in response to stress conditions, such as nutrient deprivation, may be involved in the pathology of OPMD, and highlight HNRNPQ as a potential autophagy modulator in this disease.

## 4 METHODS

### 4.1 Preparation of primary human fibroblasts

The protocols related to human tissue‐derived cells in the present study were in accordance with guidelines approved by the Helsinki committee and Institutional Review Board. Fibroblasts were extracted by a modified protocol (Ionescu et al., 2016). The OPMD patient carries a heterozygous polyalanine expansion mutation in the *PABPN1* gene. The biopsy was submerged in 1 mL collagenase II solution (MERCK, C0130) for up to 3 h at 37°C. After the incubation, a 5% BSA coated pipette tip was used to transfer the tissue to a 10 cm plate that had been briefly coated with 5% BSA and then aspirated and filled with DMEM supplemented with 2.5% Pen‐strep‐Nystatin (PSN, 03‐032‐1B, Sartorius Israel). The tissue was then transferred to another well coated with Matrigel (Corning, 354234) and BioamfTM‐2 (Sartorius Israel, 01–194‐1A) with 1% PSN was added to extract fibroblasts.

### 4.2 Cell line culture

HeLa cells were purchased from ATCC (line CCL‐2). The cells were cultured in full media, which consists of DMEM (01–052‐1A, Sartorius Israel), 10% heat‐inactivated bovine serum (FBS, 04‐007‐1A, Biological Industries) penicillin/ streptomycin (1000 μg/mL, 15240–062, Gibco Thermo Fisher Scientific), and L‐Glutamine (G7513‐100ML, MERCK). HeLa cells were cultured at 37°C in an atmosphere of 5% CO_2_ and were routinely tested for mycoplasma. For autophagy induction experiments, cells were first washed with PBS and starved in HBSS (Gibco Thermo Fisher Scientific, 14025092) for up to 4 h. In some experiments, Bafilomycin A1 (BMLCM110‐0100, Enzo Life Sciences), with DMSO (1:250, Sigma 6768‐5) as a control, were added to the cell media. Bafilomycin A1 was resuspended in DMSO to prepare a 100 μM stock solution, according to the manufacturer's specifications. MRT 68921 (Tocris, 5780) was used for pharmacological inhibition of ULK1. The compound was diluted in autoclaved water to a concentration of 10 mM. For use, the drug was further diluted 1:1000 with HBSS and incubated with the cells for 4 h.

### 4.3 Plasmids and cloning

The pEGFP‐C1 plasmids bearing the bovine PABPN1 wild type and PABPN1 + 7 Ala mutant genes were a gift from David Rubinsztein. As a first step toward deletion of the EGFP gene and insertion of the C‐terminal HA tag, we deleted the polyAla region from the wild‐type gene performing a site‐directed mutagenesis applying Inverse PCR and Q5® Site‐Directed Mutagenesis Kit (New England Biolabs, NEB). The HA‐tagged delta Ala mutant bovine gene was humanized by changing amino acids Asp 95 and Ser 102 to Ser 95 and Pro 102, respectively, applying Q5® Site‐Directed Mutagenesis Kit (NEB). Following humanization, 10 Ala (wild type) and 17 Ala (+7 mutant) regions were added using gene blocks (IDT) and Gibson assembly (NEBuilder® HiFi DNA Assembly kit, NEB). The plasmid pCMV6‐Entry‐HNRNPQ carrying the C‐terminally tagged Myc‐DDK (Flag)‐HNRNPQ transcript 1 was obtained from Origene (RC217902). The pcDNA4‐Beclin1 (FL) was a gift from Qing Zhong (Addgene plasmid # 24388; Sun et al., 2008). The ptfLC3 was a gift from Tamotsu Yoshimori (Addgene plasmid # 21074; Kimura et al., 2007).

### 4.4 Knockdown and overexpression studies

The antisense‐strand sequences against PABPN1 and HNRNPQ and control non‐targeting siRNA were purchased from Horizon. The sequences of the siRNA used are listed in Table S2. Cells were transfected with siRNAs for 48 h using Lipofectamine 2000 (11668019, Thermo Fisher Scientific) according to the manufacturer's instructions. The siRNA for PABPN1 and HNRNPQ was used at 25 nmole, with two rounds of knockdown for 5 days. Non‐targeting siRNA‐treated cells served as a control. For cDNA overexpression studies, HeLa cells or primary fibroblasts grown in six‐well plates were transfected with 0.5 μg – 1.5 μg of DNA mixed with OptiMEM (11058021, Thermo Fisher Scientific). The transfection solution was added to the cell media gently, and the cells were lysed or imaged after 24–48 h.

### 4.5 RNA isolation and RT‐PCR

Total RNA was isolated using TRIzol reagent (009010233100, Bio‐Lab) and RNeasy the Mini kit (74104, Qiagen) according to the manufacturer's protocol, and treated with DNase I (M0303S, New England Biolabs). The RIN integrity number was measured by Tape Station according to the Agilent RNA Screen Tape System. RNA was reverse transcribed to cDNA using random hexamers and Reverse Transcriptase (K1621, Thermo Fisher Scientific), with 50 ng/μl of RNA used for each reverse transcription reaction and 1 μL of cDNA employed for RT‐PCR.

### 4.6 qRT‐PCR experiments

Total RNA was extracted from cells using TRIzol according to the manufacturer's instructions. The quality and purity were determined using an ND‐1000 NanoDrop spectrophotometer, and RNA (1–3 μg) was reverse transcribed using ReverAid reverse transcriptase according to the manufacturer's instructions. The cDNA was used for the qPCR reaction using SYBR green mix buffer (PB10.22–02, PCRBIOSYSTEM) in a total of 10 μL reaction volume. Triplicate qRT‐PCR reactions were prepared using the 2x PCRBIO HS Taq Mix Red amplified with a StepOnePlus instrument (Thermo Fisher Scientific). The expression level of each mRNA was normalized to actin mRNA. Expression levels were calculated according to the ΔΔCt method. The sequences of primers used for RT‐PCR and qRT‐PCR are listed in Table S3.

### 4.7 Western blot and antibodies

Cells were washed with ice‐cold PBS and lysed by the addition of sample buffer x2. The lysates were boiled at 95°C for 3 min, and protein samples were resolved on 12%–15% SDS‐PAGE. In some experiments, samples of the cell lysates were analyzed for total protein levels using Coomassie blue staining. For western blot, proteins were transferred to 0.2 μm PVDF membranes (Bio‐Rad), which were blocked in 5% non‐fat milk in PBST (0.1% Tween 20) for 1 h at room temperature, and then incubated overnight at 4°C with primary antibodies diluted in either PBST, 5% BSA in PBST, or 5% non‐fat milk in PBST. The following primary antibodies were used: rabbit‐anti PABPN1 (ab271477, Abcam), mouse anti‐HNRNPQ (sc‐56703, Santa Cruz), mouse anti‐HA (901501, Biolegend), mouse anti‐FLAG (F1804, MERCK), rabbit anti‐ULK1 (sc‐390904, Santa Cruz), mouse anti‐UVRAG (sc‐293268, Santa Cruz), rabbit anti‐ATG14 (PD026MS, MBL), rabbit anti‐beclin 1 (3738, Cell Signaling), rabbit anti‐ATG5 (ab108327, Abcam), rabbit anti‐phospho‐beclin 1 Ser‐15 (84,966, Cell Signaling), rabbit anti‐lamin B1 (ab16048, Abcam), mouse anti‐GAPDH (5174S, Cell Signaling), rabbit anti‐actin (A2066, MERCK), and rabbit anti‐LC3B (ab192890, Abcam).

At the end of the incubation, the PVDF membranes were washed three times with PBS for 10 min. Secondary antibodies including anti‐mouse (1:10,000, ab6789, Abcam) and anti‐rabbit (1: 20,000, ab6721, Abcam) horseradish peroxidase‐conjugated secondary antibody were incubated with the membranes for 1 h in 5% non‐fat milk in PBST. ECL, a chemiluminescent Kit (XLS071, Cyanogen), was used to detect the interacting complex, and the chemical luminescence of membranes was detected using the UVITEC ALLIANCE (Uvitec, England). The intensity of protein bands was quantified by using ImageJ software.

### 4.8 Nuclear and cytoplasmic fractionation

To prepare the cytoplasmic and nuclear fractions, cells were washed twice with pre‐warmed PBSX1 and then lysed by addition of 150 μL of Buffer A (10 mM HEPES, 10 mM KCl, 0.4% NP‐40, 1 mM DTT, and protease/phosphatase inhibitors cocktail) and incubated on ice for 30 min. The homogenate was centrifuged at 16,100*g* at 4°C for 10 min, and then, 50 μL of the cytoplasmic fraction was transferred to 50 μL of Sample Buffer x4 in a pre‐labeled Eppendorf tube and the samples were boiled for 3 min at 95°C. The pellet remaining after all the cytoplasmic fraction was removed was resuspended with Buffer B (20 mM HEPES pH 7.4,10 mM KCl, 2 mM MgCl_2_, 1 mM EDTA, 1 mM EGTA, 1 mM DTT, PI cocktail III (P0044, MERCK)) and centrifuged at 900 *g* for 10 min. The supernatant was removed, and the nuclear pellet was resuspended in 100 μL of Sample buffer x2, and boiled for 3 min at 95°C.

### 4.9 RNA immunoprecipitation (RIP)

The protocol for RIP was as described previously (Keene et al., 2006). Briefly, agarose beads (Santa Cruz, Biotechnology, Inc.) were resuspended in NT2 buffer (50 mM Tris–HCl pH 7.4, 150 mM NaCl, 1 mM MgCl_2_, 0.05% NP‐40 (11754599001, MERCK) with 1× protease inhibitor cocktail and 5% of BSA (A7030, MERCK). The samples were incubated overnight at 4°C together with the primary antibody mouse anti‐HNRNPQ or rabbit anti‐PABPN1 or isotype unrelated control antibodies, with gentle rotation, before being washed and resuspended in 850 μL of NT2 buffer. After 4 h starvation, HeLa cells were washed once with pre‐warmed PBS supplemented with calcium and magnesium before 1 mL of trypsin was added and then neutralized with 3 mL of full media. The cell suspension was centrifuged at 2000 *g*, 4°C for 5 min, washed with cold PBS with calcium and magnesium, and centrifuged again to remove the PBS. After this step, the cell pellet was gently resuspended in Polysome lysis buffer (PLB): (150 mM KCl, 25 mM Tris–HCl pH 7.4, 5 mM EDTA, 0.5% NP‐40, 0.5 mM DTT, supplemented with 100 U/mL RNAase inhibitor (3335399001, MERCK) and 1× protease inhibitor cocktail) and incubated on ice for 15 min before centrifugation at 14,000 *g* at 4°C for 10 min in order to harvest the supernatant. An aliquot (10%) of the lysate was frozen at −80°C until used for analysis of total RNA. The remainder (150 μL) of lysate was transferred to prepared tubes containing beads and antibody, and the samples were incubated overnight at 4°C on a rotator device. The manufacturer's instructions were followed for RNA extraction.

### 4.10 RNA‐seq analysis and bioinformatics

In short, a NEBNext Ultra II Directional RNA Library was prepared with the NEBNext Poly(A) mRNA Magnetic Isolation Module from Control (n = 3), HNRNPQ (n = 3) and PABPN1 (n = 2) RIP experiments. The library was amplified by 12 PCR cycles, with an additional bead‐based clean‐up at the end of the protocol (x0.8) to remove adapter‐dimers. Libraries were sequenced on a NextSeq 500 instrument using a NextSeq 500/550 High Output Kit v2.5 (75 Cycles) kit. FastQ files were analyzed using Partek® Flow® build version 10.0.21.1116 (https://www.partek.com/partek‐flow/). Reads were trimmed to remove low quality bases from the 3′ end (Phred <20). Alignment performed using STAR 2.7.8a to human genome (GRCh38; Dobin et al., 2013). The reads were quantified using Partek E/M (Xing et al., 2006) (27,805 genes detected) with DESeq2 median ratio normalization and statistical analysis (Love et al., 2014). Differentially expressed genes (DEGs) with *p*‐value cutoff *p* < 0.05 and fold‐change (FC) difference >0 between each treatment and control were identified and the Venny tool (https://bioinfogp.cnb.csic.es/tools/venny/index.html) was used to generate a Venn diagram of known genes (excluding non‐coding RNAs). The function enrichment of the identified DEGs was analyzed using DAVID (Sherman et al., 2022; v2022q3 release).

### 4.11 Immunostaining and imaging

Cells were grown on coverslips and were washed twice with pre‐warmed PBS and fixed in PBS 4% formaldehyde. They were then washed three times with PBS and incubated with 0.1% Triton X100 in PBS. After another three washes in PBS, the cells were blocked in PBS 1% BSA and incubated with primary antibodies diluted at 1:150 in 1% BSA for at least 1 h or overnight. After this time, the cells were washed once with 1% BSA and twice with PBS, and then, the coverslips were incubated with the appropriate secondary antibody (Alexa Fluor 488 or 555, Abcam) diluted 1:300 in 1% BSA for 20 min before repetition of the previous washing procedure. The washed coverslips were incubated with DRAQ5 (65–0880‐92, Thermo Fisher Scientific) diluted at 1:2000 in PBS for 5–30 min while shaking and then mounted onto microscope slides using mounting medium. The PROTEOSTAT® Aggresome detection kit (Enzo Life Sciences, # ENZ‐51035) was used according to the manufacturer's protocol to detect aggregates in fibroblasts (Wrobel et al., 2022). The slides were allowed to dry for up to 24 h at room temperature and then stored at 4°C until they were imaged on a Zeiss 710 confocal microscope. Colocalization of PABPN1 and HNRNPQ in the nucleus was measured using the imageJ plugin Coloc2. Images of HeLa cells transfected with different HA‐tagged PABPN1 constructs were analyzed with or without starvation treatment by using the imageJ plugin, and considering only areas in the nuclei of successfully transfected cells. A Pearson's correlation coefficient was generated for various image fields. In some experiments, ULK1 intensity (using rabbit anti‐ULK1 antibody, ab240916 Abcam) was measured by circulating the FLAG‐HNRNPQ positive patient cells and control cells. The intensity was measured for the channel of interest, and the mean value and the area of each circulated cell were recorded using Fiji. The value of the mean divided by the area was used for statistical analysis.

### 4.12 Statistical analysis

Results are expressed as the mean ± SEM. ANOVA or *t* tests were used for statistical analysis. Statistical analysis was performed using GraphPad Prism 8.

## AUTHOR CONTRIBUTIONS

Avraham Ashkenazi, Hasan Ishtayeh, Margarita Galves, and Tania T. Barnatan wrote the manuscript with inputs from all authors. Hasan Ishtayeh, Margarita Galves, Tania T. Barnatan, and Fatima Amer‐Sarsour performed and analyzed cell experiments. Sergiu C. Blumen and Itzhak Braverman recruited patient and obtained patient biopsies. Metsada Pasmanik‐Chor performed RNA‐seq analysis and bioinformatics. Yevgeny Berdichevsky performed molecular biology experiments. Avraham Ashkenazi supervised the study.

## CONFLICT OF INTEREST STATEMENT

The authors declare that they have no conflict of interest.

## Supporting information


Data S1.

Data S2.

## Data Availability

Raw fastq files of the RIP experiment were uploaded to GEO (accession code GSE222017).
