# Supplementary material for: Oculopharyngeal muscular dystrophy mutations link the RNA‐binding protein HNRNPQ to autophagosome biogenesis
Source: Aging Cell. 2023 Aug 9;22(10):e13949. doi: 10.1111/acel.13949 (PMC10577562; doi:10.1111/acel.13949)
Supplement: Supplementary file 1 — Data S1. [file ACEL-22-e13949-s001.pdf]

Supplementary Information

OPMD disease mutations link the RNA binding protein HNRNPQ to autophagosome biogenesis

Supplementary Tables

Table S1. List of genes from the RIP experiment enriched in the brown and pink modules (related to Fig. 2, enclosed xls. file).

Table S2. List of siRNAs used in this study.

siRNA	Sequence
Non-targeting pool	UGGUUUACAUGUCGACUAA
	UGGUUUACAUGUUUUCUGA
	UGGUUUACAUGUUGUGUGA
	UGGUUUACAUGUUUUCCUA
PABPN1 siRNA 5	GGAACGGCCUGGAGUCUGA
PABPN1 siRNA 6	AGUCAACCGUGUUACCAUA
HNRNPQ siRNA 5	GCACAUAGUGAUUUAGAUG
HNRNPQ siRNA 6	GAGGUUAUGGCAAAGGUAA
HNRNPQ siRNA 7	GUAGAGGUGGUUAUGGAUA
HNRNPQ siRNA 8	GUUAUGCGUUUGUCACUUU

Table S3. List of primers used in the qRT PCR analysis.

Gene name	Forward	Reverse
β-Actin	AGAGCTACGAGCTGCCTGAC	AGCACTGTGTTGGCGTACAG
ATG14	CGACCGGGAGAGGTTTATCG	TCAATCCTCATCTTGCAGGACAT
ULK1	CCTCGGTCAGGAAATCCCCA	CTTGCTGCCACCAGAGTCCC
UVRAG	AGCGGCGTCTTCGACATCTT	TCGCCACGTGGGATTCAAGG

Supplementary Figures

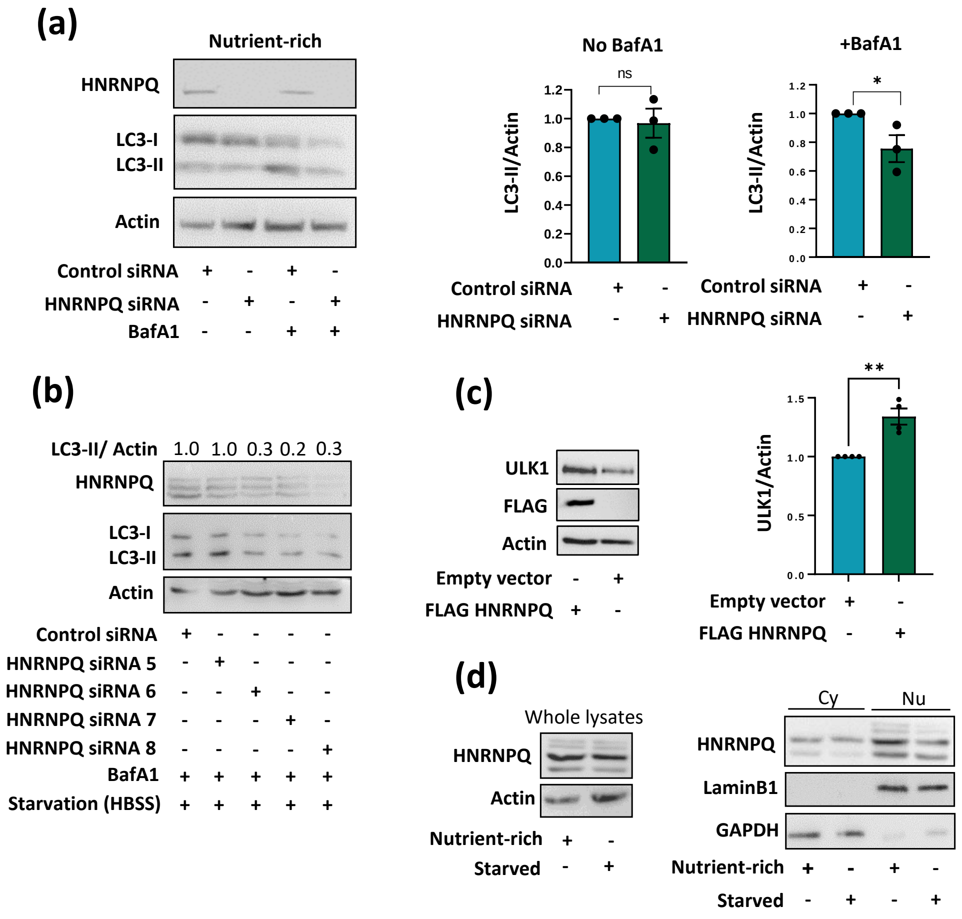

Figure S1. Effects of HNRNPQ depletion on autophagy. (a) HeLa cells were treated with siRNA targeting HNRNPQ or control siRNA. The cells were incubated with or without exposure to BafA1 (250 nM, 4 h in nutrient-rich media). Cell lysates were analyzed for LC3-II levels. Results are normalized to control cells: n=3 experiments, paired two-tailed *t*-test, * $p < 0.05$, ns is non-significant. (b) Control and different siRNAs targeting HNRNPQ were incubated with the cells, and 4 h prior to lysis, the cells were treated with HBSS together with BafA1. Cell lysates were analyzed for HNRNPQ and LC3-II levels (ratio of LC3-II/actin is presented). (c) Cells were transfected with FLAG HNRNPQ or with a control empty vector and starved (HBSS, 4 h). Cell lysates were analyzed for ULK1 levels. Results are normalized to control cells: n=4 experiments, paired two-tailed *t*-test, ** $p < 0.01$. (d) Cells were starved (HBSS, 4 h) or kept in nutrient-rich media. The nuclear (Nu) and cytoplasmic (Cy) fractions were isolated and probed for HNRNPQ, LaminB1 (nuclear marker), and GAPDH (cytoplasmic marker).

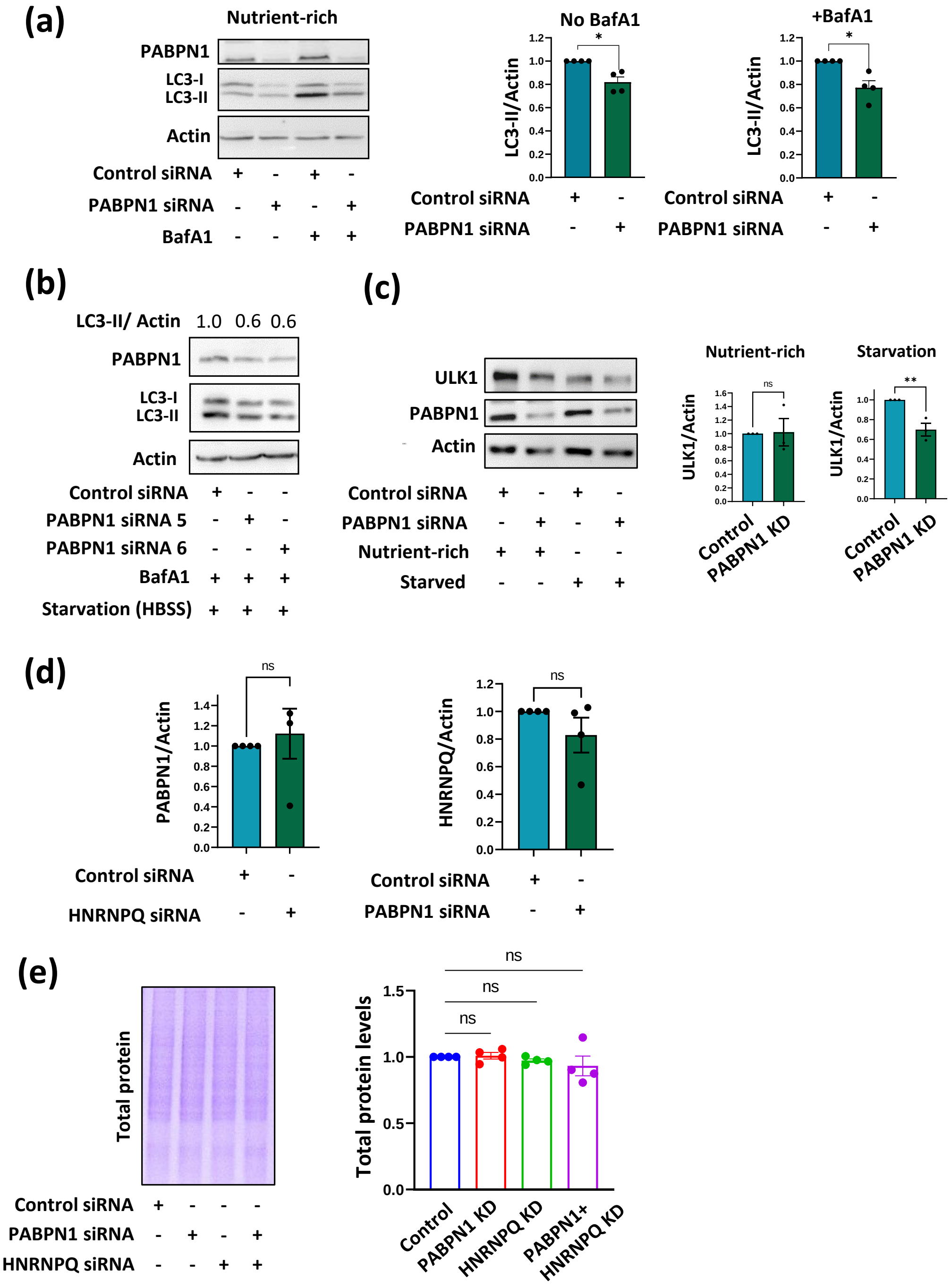

Figure S2. PABPN1 regulates autophagosome formation. (a) HeLa cells were treated with siRNA targeting PABPN1 or control siRNA. The cells were incubated with or without BafA1 (250 nM in nutrient-rich media for 4 h). Cell lysates were analyzed for LC3-II levels. Results are normalized to control cells: n = 4 experiments. (b) Control and different siRNAs targeting PABPN1 were incubated with the cells, and 4 h prior to lysis, the cells were treated with HBSS together with BafA1. Cell lysates were analyzed for PABPN1 and LC3-II levels (ratio of LC3-II/actin is presented). (c) PABPN1 KD and control cells were cultured in nutrient-rich media or starved, and

cell lysates were analyzed for ULK1 levels. Results are normalized to control cells: n = 3 experiments. (d-e) d, Detection of HNRNPQ and PABPN1 protein levels in starved control cells, HNRNPQ KD cells, and PABPN1 KD cells (representative blots are shown in **Fig. 2c**). e, Quantification of total protein levels in HNRNPQ and PABPN1 KD cells. n = 4 experiments. Paired two-tailed *t*-test. * $p < 0.05$, ** $p < 0.01$, ns is non-significant.

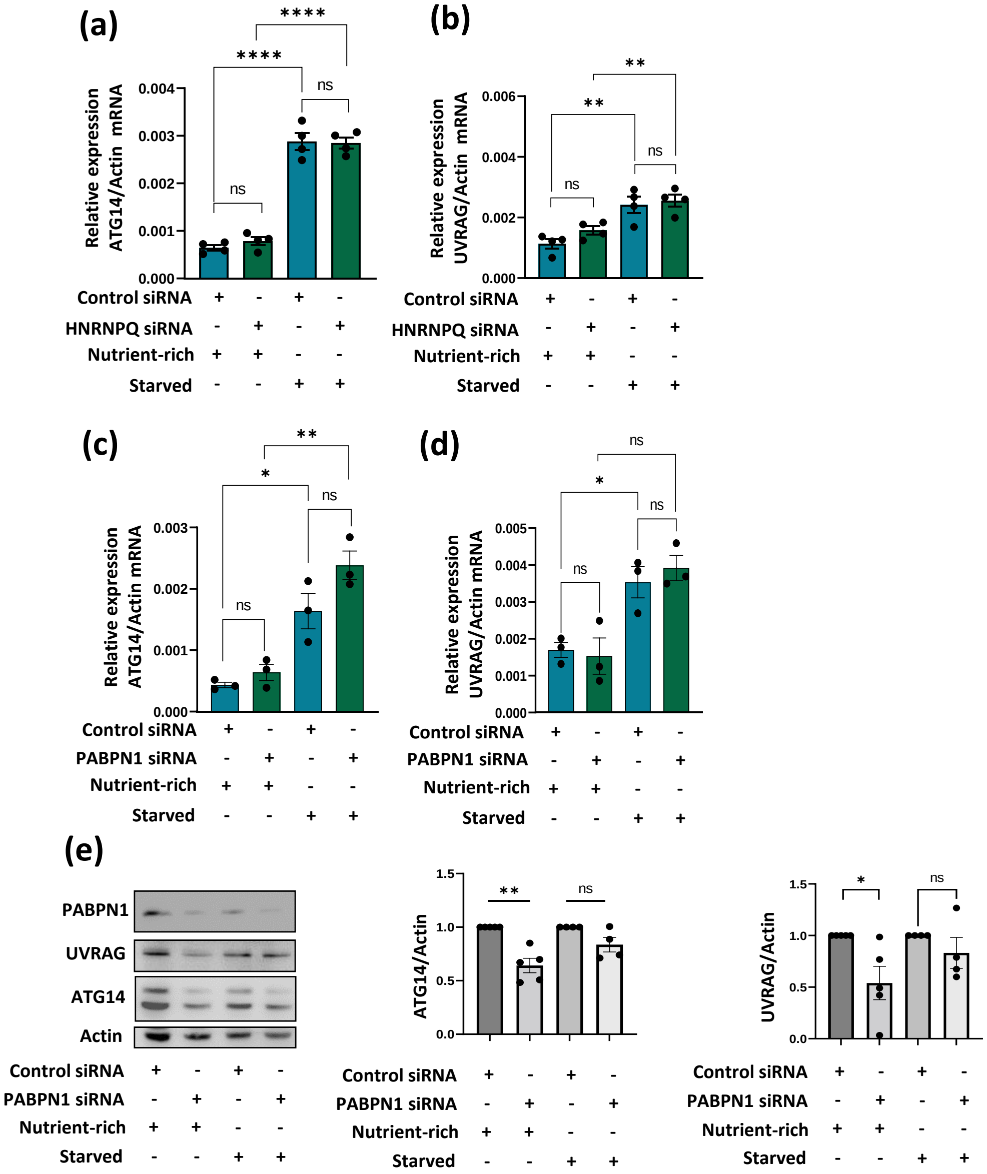

Figure S3. Effect of PABPN1 and HNRNPQ on the levels of ATG proteins in beclin 1 complexes. (a-b) Analysis of ATG14 and UVRAG mRNA levels by qRT-PCR in control and HNRNPQ KD HeLa cells under nutrient-rich and starvation conditions: n = 4 experiments. (c-d) Analysis of ATG14 and UVRAG mRNA levels by qRT-PCR in control and PABPN1 KD HeLa cells under nutrient-rich and starvation conditions: n = 3 experiments. (e) PABPN1 KD and control cells were cultured in nutrient-rich media or starved, and cell lysates were screened for ATG14 and UVRAG. ATG protein levels are normalized to actin. Results are from at least four experiments. Paired two-tailed *t*-test. * $p < 0.05$, ** $p < 0.01$, **** $p < 0.0001$ ns is non-significant.

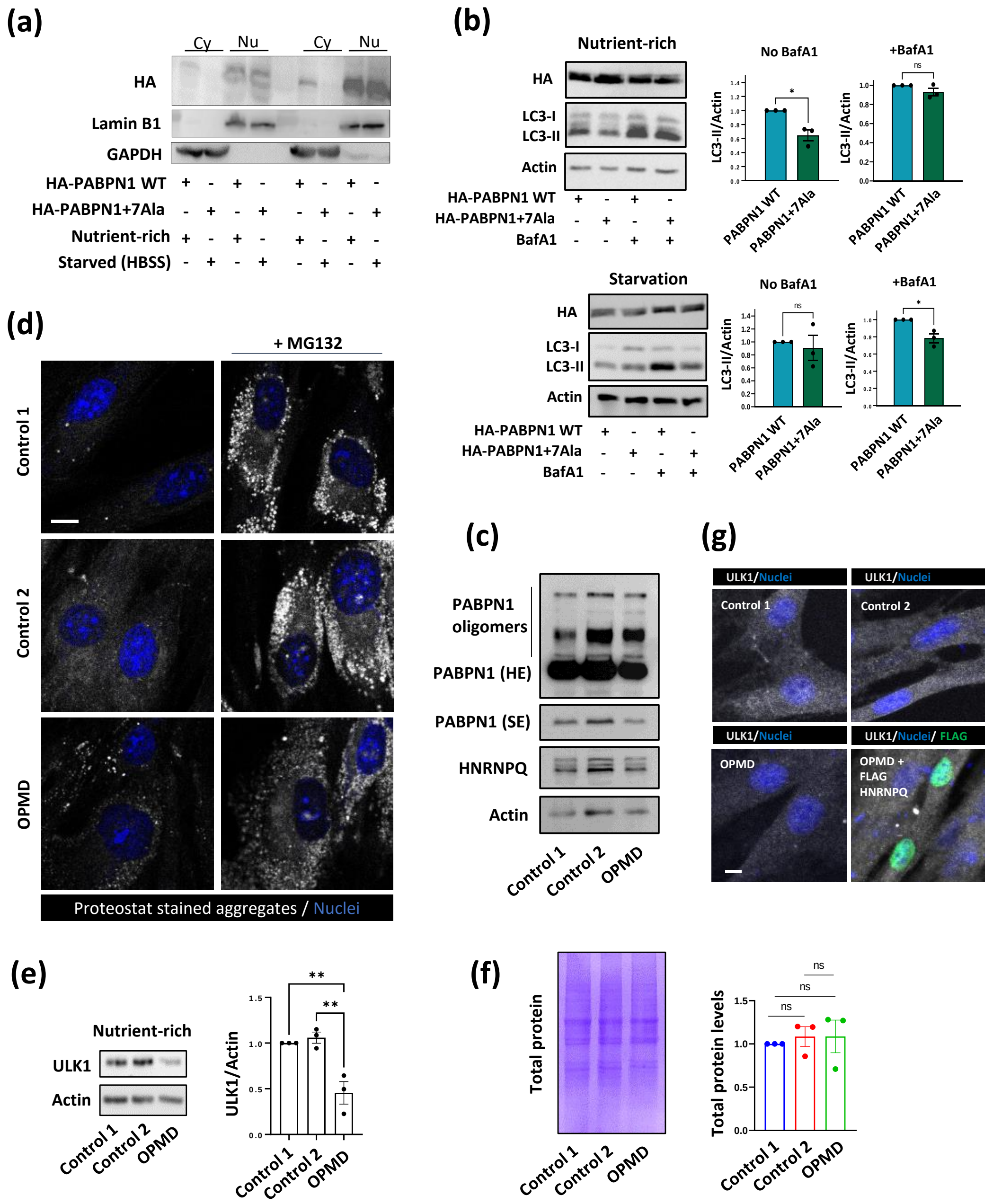

Figure S4. PABPN1 cellular localization, oligomerization, and induction of misfolded protein accumulation in mutant PABPN1 expressing cells. (a-b) HA-PABPN1 WT or HA-PABPN1 mutant (+7 Ala) were expressed in HeLa cells. (a) Cells were starved (HBSS, 4 h) or maintained in nutrient-rich media. The nuclear (Nu) and cytoplasmic (Cy) fractions were isolated and probed for HA, LaminB1, and GAPDH. (b) Cells maintained in nutrient-rich condition or starved with or without BafA1 (250 nM, 4 h), and cell lysates were analyzed for LC3-II levels. Results (n = 3 experiments) are normalized to control cells. Paired two-tailed *t*-test. (c) Control and OPMD patient-derived fibroblasts were lysed and analyzed for SDS-resistant oligomers of PABPN1. (d) Control and OPMD fibroblasts were treated with or without MG132 (10 µM, 6h). Cells were fixed

and stained with Proteostat dye. Representative images of the Proteostat staining are shown (colored gray). (e-f) Control and OPMD patient fibroblasts were analyzed for ULK1 levels (e), and for total protein levels (f). Results are normalized to control fibroblasts. n = 3 experiments. One-way ANOVA Tukey’s test. (g) Images of ULK1 staining in control and OPMD patient fibroblasts that were transfected with FLAG HNRNPQ (quantification is shown in Figure 4d). Scale bar 10 µm. * $p < 0.05$, ** $p < 0.01$, ns is non-significant.
